# Correction for measurement error in invariance testing: An illustration using SQP

**DOI:** 10.1371/journal.pone.0239421

**Published:** 2020-10-01

**Authors:** André Pirralha, Wiebke Weber

**Affiliations:** 1 RECSM, Universitat Pompeu Fabra, Barcelona, Spain; 2 Sociometric Research Foundation, Barcelona, Spain; University of Copenhagen, DENMARK

## Abstract

With the increasing availability of cross-national data, more attention has been given to the issue of comparability. But while a lot of emphasis has been directed to the assessment of measurement invariance, there has been substantially less concern on how measurement error can affect the results of measurement invariance testing. In this study, we show how correction for measurement error can be applied to measurement invariance analysis. We illustrate this using the concept of “Perceived ethnic threat” measured in the European Social Survey Round 3 (2006). The measurement invariance results before and after correction for measurement error are compared. We show that correction for measurement error offers a viable way to ensure that non-invariant parameters are actually caused by differences in the data and not caused by the measurement method.

## 1. Introduction

In recent years European societies have experienced deep social changes often connected to immigration. More pronouncedly, Western Europe has been an attractive destination for large-scale immigration since the Second World War and recent research has focused on the substantial migration inflows that developed in the last decades. The increase of immigrant population has changed the ethnic configuration of European countries and affected perceptions regarding immigrant groups [1]. There is evidence that a considerable fraction of the European population does not look at immigration with favorable eyes but considers it as a threat to the economic, social and political order, the cultural homogeneity and the national identity [2]. This fact is particularly visible in the electoral growth of anti-immigration parties in several European countries like the United Kingdom, France, Denmark or the Netherlands [3]. Several studies have focus on the origins of the perception of immigration as a threat. The Group Conflict Theory is probably one of the most significant approaches, conceptualizing prejudice and anti-immigration perceptions as defensive reactions to the insight of intergroup competition for conflicting goals and/or over limited resources [2, 4, 5]. It has been well established that socio-economic characteristics, such as income, education and unemployment, are strongly related with perceptions of immigration as an ethnic threat [6–8]. Further research has also started to take into consideration eventual contextual effects such as the quantitative demographic relation between groups and the amount of resources for which the groups are competing [9, 10]. In order to investigate these effects, the field of (anti-) immigration attitudes took advantage of the growing number of available cross-national data sources, in what has been characterized as the “cross-national turn”, and made comparing between countries a fundamental element to test the validity of the theory [11]. However, though cross-national comparisons bring along great advantages it is not without challenges: before cross-national comparisons can be made, measurement equivalence between countries has to be established. Not doing so can lead to biased and not meaningful comparisons between different groups or countries.

Measurement equivalence, also referred to as measurement invariance, was defined by Horn and McArdle [12, pp. 117] as whether “under different conditions of observing and studying phenomena, measurement operations yield measures of the same attribute”. In other words, measurement equivalence is a property of the measurement instrument, indicating that it measures the same concept in the same way across different groups [13]. Of course, this does not mean that all and any differences between groups should be seen as deviant. Instead, if measurement invariance holds, it implies that differences across groups in a given concept should be attributable only to cross-cultural differences in the constructs and not to measurement artifacts. On the other hand, if the answers to a measurement instrument are influenced by additional factors other than the differences in the construct, then the results are to be considered not invariant or non-equivalent [14].

There is growing awareness of the importance of testing the measurement invariance assumption not only regarding immigration attitudes but also other subjects such as political trust [e.g. 9, 15] or universal human values [e.g. 16–19]. However, there has been significant less concern on how measurement error can affect the substantive conclusions of measurement invariance testing, although there is vast evidence that measurement error is an important issue in survey research [20, 21]. Even though random measurement error is usually included in the invariance analysis models by using multiple indicators and structural equation modeling [22], systematic measurement error, on the contrary, is often disregarded and usually not accounted for when it comes to invariance testing [23]. However, if the size of the systematic error is different across the groups under comparison, the conclusions regarding measurement invariance can be biased by assuming that the groups have equal systematic error. While some procedures have been developed to deal with this issue [24], this paper presents an alternative approach which allows correcting for random and systematic measurement error. We illustrate this with the concept of “perceived ethnic threat” as measured in the European Social Survey (ESS) Round 3 (2006). In this paper we aim to: (1) determine if the attitudes towards immigration captured by the concept “perceived ethic threat” measured in the ESS are measurement invariant across countries and (2) assess to which extent correction for measurement error yields different conclusions regarding invariance. This is, as far as we can tell, the first research study in which measurement invariance testing with correction for measurement error is compared with the standard approach.

## 2. Measurement invariance and bias

Survey research is fundamentally based on the assumption that all respondents have the same response function. This means that two respondents with the same opinion regarding any given subject will select the same answer category [25]. However, if respondents interpret the question or the response categories differently, then there is the chance that they might choose different answer options even if their opinion regarding the subject is the same. Testing for measurement invariance is the procedure to assure that the fundamental assumption of the response function being the same for all respondents is actually fulfilled. Another way to understand measurement invariance is to focus on what can actually cause non-invariance. The bias framework developed in the field of cross-cultural studies is particularly useful to this end. In order to systemize the sources of error that challenge cross-cultural comparisons, three different types of bias can be distinguished: construct bias, method bias, and item bias [26]. The construct bias exists when a construct differs across cultures. An example can be found in the work of Chen et al. [27] in the study of the cultural meaning of perceived control. While studying individualistic and collectivistic cultures, the authors found that in the first set of cultures the external locus of control carries a negative connotation while in the second it is viewed as positive. The second element of the bias framework is method bias which refers to methodological and procedural aspects of survey research. Van de Vijver [26] further divides method bias into three further types: sample, administration, or instrument. Examples of such sources of disturbance can be background characteristics of samples, interviewers and test administrators influence, and measurement instrument properties. Last, item bias regards anomalies at the item level. This can be caused by several factors such as poor item translation or item wording, as well as group-specific nuisance factors or item word connotations.

Independent of the kind of bias, what they have in common is they can cause measurement non-invariance and have therefore implications in the comparability of survey data. That is the reason why testing for measurement invariance is very important rather than just assuming it is given. Measurement invariance testing under the Multi-Group Confirmatory Factor Analysis (MGCFA) bottom-up approach framework consists in testing several progressively constrained levels of invariance. The alternative is a top down approach (see [56]). Each level of invariance corresponds to a more constrained MGCFA model. The first and least strict level is configural invariance which means that the factorial structure is the same for all groups [28, 29]. It implies therefore identical patterns of correlations and the absence of cross-loadings. The subsequent level of invariance testing, metric invariance, requires that the factor loadings of the indicator variables are equivalent across the groups. If this level of invariance is proven to hold, the unstandardized relationships between the latent construct and any other variables can be meaningfully compared, allowing the comparison of estimated factor variances and covariances across groups since it is known that the factor explains the same amount of variance in item responses.

Finally, the third level, scalar invariance, adds to the equality of factor loadings the condition of equality of the intercepts. When scalar invariance is established, the latent variable means can be meaningfully compared across groups. When all the factor loadings and intercepts are invariant, full scalar invariance has been reached. Some authors test for even more restricted models (e.g. equal residual variances known as strict factorial invariance). However, because researchers in the social sciences are more often interested in comparing the scores of the latent variable across groups, we only discuss the configural, metric and scalar levels of invariance. Finally, a small note regarding partial invariance should be added. Several authors suggest that even when the parameters are not all invariant, meaningful comparisons can still be drawn across groups [30]. When at least two out of three items per construct are invariant, partial invariance is established and comparisons across groups are still valid [31].

## 3. Correction for measurement error

Measurement error is the difference between the observed answer and the actual unobserved opinion of the respondent. More precisely, measurement error is here defined as the counterpart of measurement quality (q^2^) and consists of a random and a systematic part. Random measurement error (e) will always occur and can be due to misunderstandings of the question or answer scale, typing or coding error, etc [32]. Systematic measurement error, on the other hand, can be a reaction to the method (M). For instance, the reaction to the length of a response scale or the presence of an interviewer. In Fig 1 we present the measurement model of the variable of interest (f). The relationship between this variable of interest and the observed variable (y) can be decomposed into the stable component, the true score (t), the systematic method factor (M) and the random error (e). The validity coefficient (v) squared is the validity (v^2^) which indicates the strength of the relationship between the variable of interest and the true score, while the squared reliability coefficient (r), the reliability (r^2^) indicates the relationship between the true score and the observed variable. Saris and Gallhofer [22] define measurement quality (q^2^) as the product of reliability (r^2^) and validity (v^2^).

**Fig 1:**
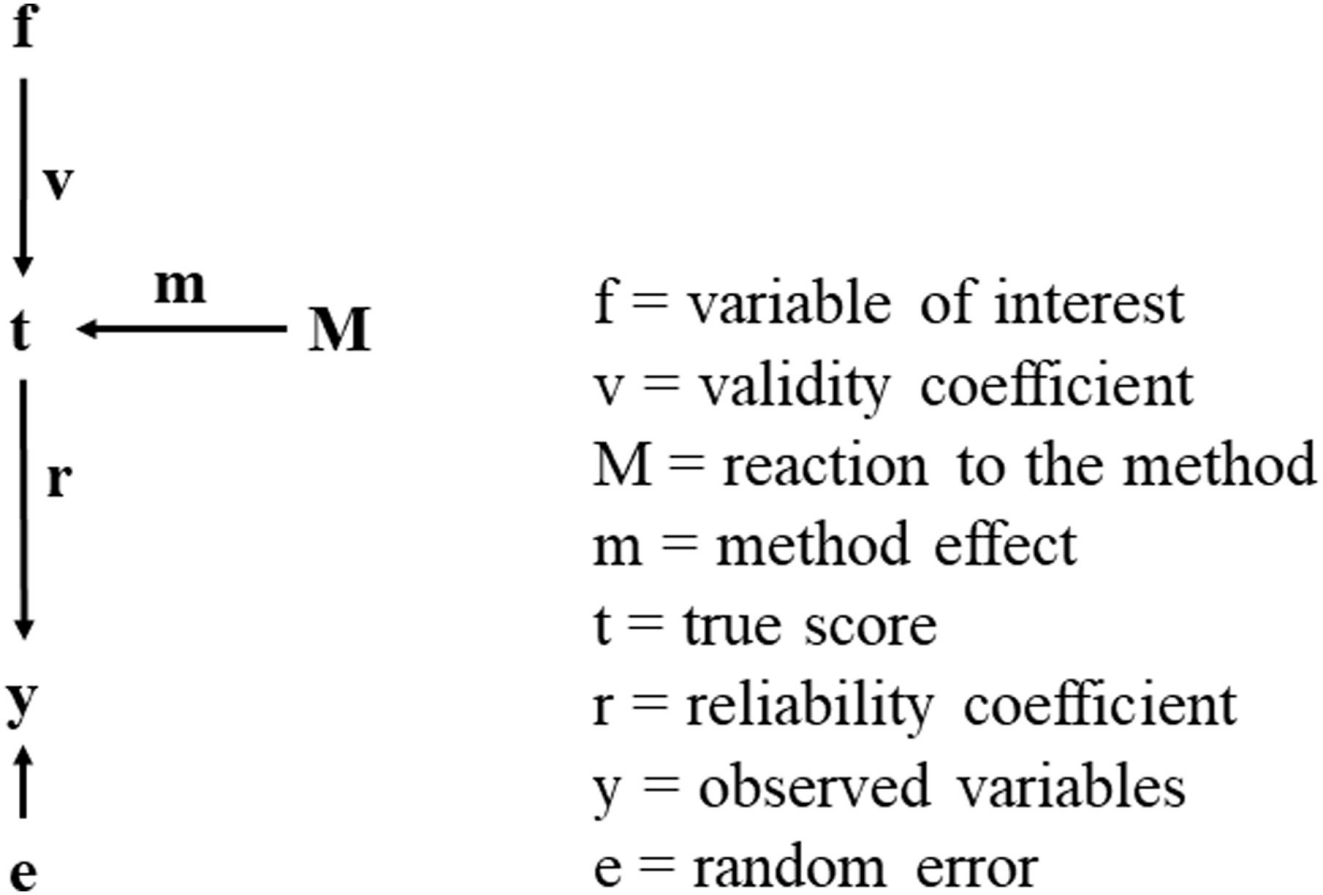
Measurement model with systematic method factor (M) and random error (e). Source: Saris & Gallhofer (2014), Fig 9.6, page 176.

The measurement quality ranges from 0 to 1, following the cut-off points from Cronbach’s alpha we can say that a q^2^ < .6 is poor, 0.6< q^2^ ≤.7 is questionable, 0.7< q^2^ ≤.8 acceptable, 0.8< q^2^ ≤.9 good, and q^2^ ≥ 0.9 is excellent quality [33].

For a long time, the main obstacle for correction of measurement error was the lack of information about the size of these errors. There are different ways to estimate the measurement quality [20], but they need to be implemented at the time of data collection, normally being costly and time-consuming, with the clear disadvantage that the estimates of particular questions cannot be used for other questions. Therefore, Saris and colleagues [34, 35] provided a way to overcome this problem by predicting the measurement quality based on a meta-analysis of 3,726 questions which were part of Multitrait-Multimethod (MTMM) experiments in more than 20 languages and the coding of the characteristics of the questions included in those experiments. The free license Survey Quality Predictor (SQP) software [36] does contain this information for more than 16,000 questions and allows coding new questions in order to predict their measurement quality. As an open source research project, anyone can contribute to the SQP database, include their own question and coding and/or code other user’s questions. This prediction has a R^2^ of 0.84 for the validity coefficient (v) logits and 0.65 for the reliability coefficient (r) logits [35]. Although SQP does not yet predict the measurement quality perfectly, it is, to our knowledge, the only software that contains this kind of information. Most studies do not account for measurement error at all and thus assume a perfect relationship between the observed variable and the variable of interest, i.e. measurement quality equals one. In this paper, while we rely on SQP predictions, we are fully aware that these are not perfect and measurement quality might be under- or overestimated. However, at the very least, accounting in some way for measurement error will probably bring us closer to true values rather than assuming our measures are perfect. Thus, with the prediction or estimates of measurement quality, correction for measurement error is possible and invariance testing can and should be performed after correction for differences in the measurement process [22].

## 4. Measurement invariance with correction for measurement error

As discussed before, measurement non-invariance can be caused by differences in the construct or by method differences that can be originated, amongst other things, by the measurement instruments [22]. Between these two causes of non-invariance, construct differences cannot be corrected, it simply means that the groups have different understandings of the construct and cannot be compared. On the other hand, the differences in measurement across the groups can be represented in the measurement equation and corrected. Saris and Gallhofer [22] demonstrated that for meaningful comparisons only the cognitive and not the measurement part have to be invariant. By cognitive part the authors refer to how respondents cognitively react to the stimuli, in contrast with the measurement part, which refers to the way in which respondents answer to the question. When the cognitive and the measurement process are separated, invariance only needs to be tested for the cognitive part, as the measurement part can be corrected by using measurement quality predictions. This is where this approach distinguishes itself from other invariance testing approaches. Already Little (2013: 143), showing his disagreement with strict factorial invariance testing, argued that if the sum of indicator specific and random error is not exactly equal across groups, this would create problems to other estimated parameters of the model. If systematic error is not explicitly included in the model it can potentially inflate the concept of interest.

In Fig 2 we present a factor model with three indicators for invariance testing with correction for measurement error. Using the notation presented by Saris and Gallhofer [22], in this model we have at the top the concept-by-postulation (F), operationalized by the concepts-by-intuition f_1_, f_2_ and f_3_. The measurement quality (q^2^) is defined as the product of reliability (r^2^) and validity (v^2^), and the quality coefficient (q) is the product of the reliability coefficient (r) and validity coefficient (v). As the coefficients for the measurement part, i.e. the reliability coefficient (r), validity coefficient (v), and the effect of the method (m), can be obtained through other means (therefore here illustrated in grey and the corresponding effects with dashed arrows), only the upper part of the model needs to be estimated and invariance is tested by the standard procedure of constraining the loadings (c_i_) of the cognitive part to be equal across groups.

**Fig 2:**
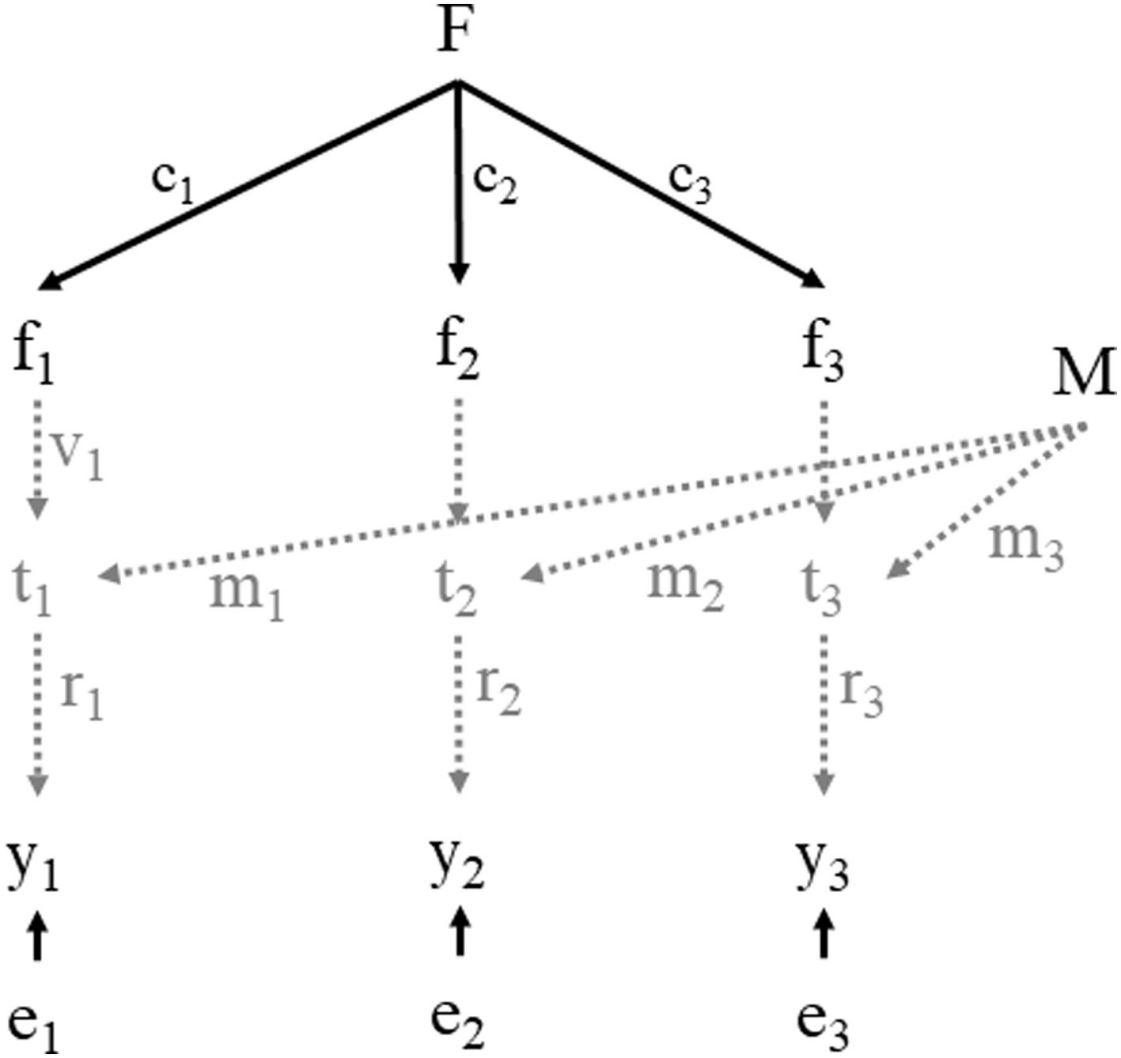
Factor model with three indicators and the separation of cognitive and measurement part.

## 5. Data

The data we use to exemplify measurement invariance testing with correction for measurement error are the three variables that measure the “Perceived ethnic threat” concept in the ESS Round 3 (2006) [37]. Respondents are asked to state their opinion about immigrants’ impact on the country’s economy, cultural life, and life in general. Table 1 presents the wording and the 0 to 10 item-specific response scale of the questions with the respective reference points.

**Table 1:** Questions wording and response scales for the “Perceived ethnic threat”.

Variable name	Question	Response scale
“economy”	Would you say it is generally bad or good for [country]’s economy that people come to live here from other countries?	0 bad for the economy– 10 good for the economy
“culture”	Would you say that [country]’s cultural life is generally undermined or enriched by people coming to live here from other countries?	0 cultural life undermined– 10 cultural life enriched
“better”	Is [country] made a worse or a better place to live by people coming to live here from other countries?	0 worse place to live– 10 better place to live

For the measurement quality predictions we rely on the information already available in SQP2.1 as authorized predictions. SQP allows coding own questions and obtaining a quality prediction but also to consult other users’ codings and predictions as well as authorized codings and predictions. In this paper we use only authorized predictions. The difference is that they have been coded by native speakers, trained and supervised by the SQP team. The coding scheme of SQP is well defined, covers up to 60 characteristics depending on the survey question, of which only few are in fact subjective and thus up to the coder’s decision [38]. The developers of SQP have therefore decided not to present intercoder reliability but rather followed a team approach of review and adjudication, as suggested in other research fields such as survey translation [39]. While complete information about SQP 2.1 can be found elsewhere [36], for readers’ convenience we present in Annex I the SQP authorized coding of the 54 characteristics of the “economy” question as measured in the ESS.

Relying on authorized predictions has the advantage that we are sure that the codifications of the question’s characteristics are correct but has the disadvantage that the predictions are only available for 19 country groups in ESS Round 3 [37]. As SQP is an ongoing research project open for collaboration of the academic community and the SQP users, the availability of the data depends on this collaboration. In Table 2 we present for each question in the 19 country groups its quality coefficient (q_i_), which is the product of validity coefficient (v_i_) and the reliability coefficient (r_i_), that can be directly obtained by SQP2.1. Moreover, we also present the effect of the method (M) on the observed variables (y_i_) which is the product of the reliability coefficient (r_i_) and the method effect coefficient (m_i_). The latter is calculated as follows: *m*_*i*_ = *r*_*i*_*μ*_*i*_, where $\mu_{i}\;=\;\sqrt{\left(1-v_{i}^{2}\right)}$.

**Table 2:** SQP authorized quality predictions for indicators/questions.

Country	economy		culture		better	
	q	m	q	m	q	m
Austria	0.786	0.415	0.743	0.437	0.759	0.430
Belgium	0.799	0.379	0.766	0.434	0.756	0.374
Denmark	0.831	0.346	0.778	0.423	0.781	0.413
Estonia	0.792	0.391	0.735	0.430	0.753	0.445
Finland	0.803	0.388	0.769	0.462	0.765	0.423
France	0.779	0.407	0.753	0.439	0.756	0.433
Germany	0.792	0.384	0.750	0.421	0.765	0.407
Ireland	0.775	0.412	0.733	0.473	0.737	0.453
Netherlands	0.838	0.317	0.801	0.368	0.799	0.298
Norway	0.788	0.419	0.758	0.457	0.753	0.461
Poland	0.786	0.407	0.739	0.441	0.734	0.444
Portugal	0.807	0.426	0.755	0.450	0.760	0.459
Slovakia	0.774	0.407	0.716	0.447	0.708	0.437
Slovenia	0.778	0.403	0.722	0.424	0.721	0.435
Spain	0.799	0.396	0.747	0.443	0.742	0.426
Sweden	0.778	0.408	0.736	0.441	0.728	0.431
Switzerland	0.815	0.347	0.798	0.385	0.809	0.292
Ukraine	0.778	0.435	0.704	0.489	0.728	0.486
United Kingdom	0.782	0.383	0.739	0.438	0.756	0.415

q = quality coefficient, m = method effect coefficient.

We introduce the quality coefficient (q) and method effect coefficient (m) as fixed values for each observed variable in the model for each country group. In Fig 3 we illustrate this for the case of Austria. The model can then be estimated and the results will be corrected for measurement error.

**Fig 3:**
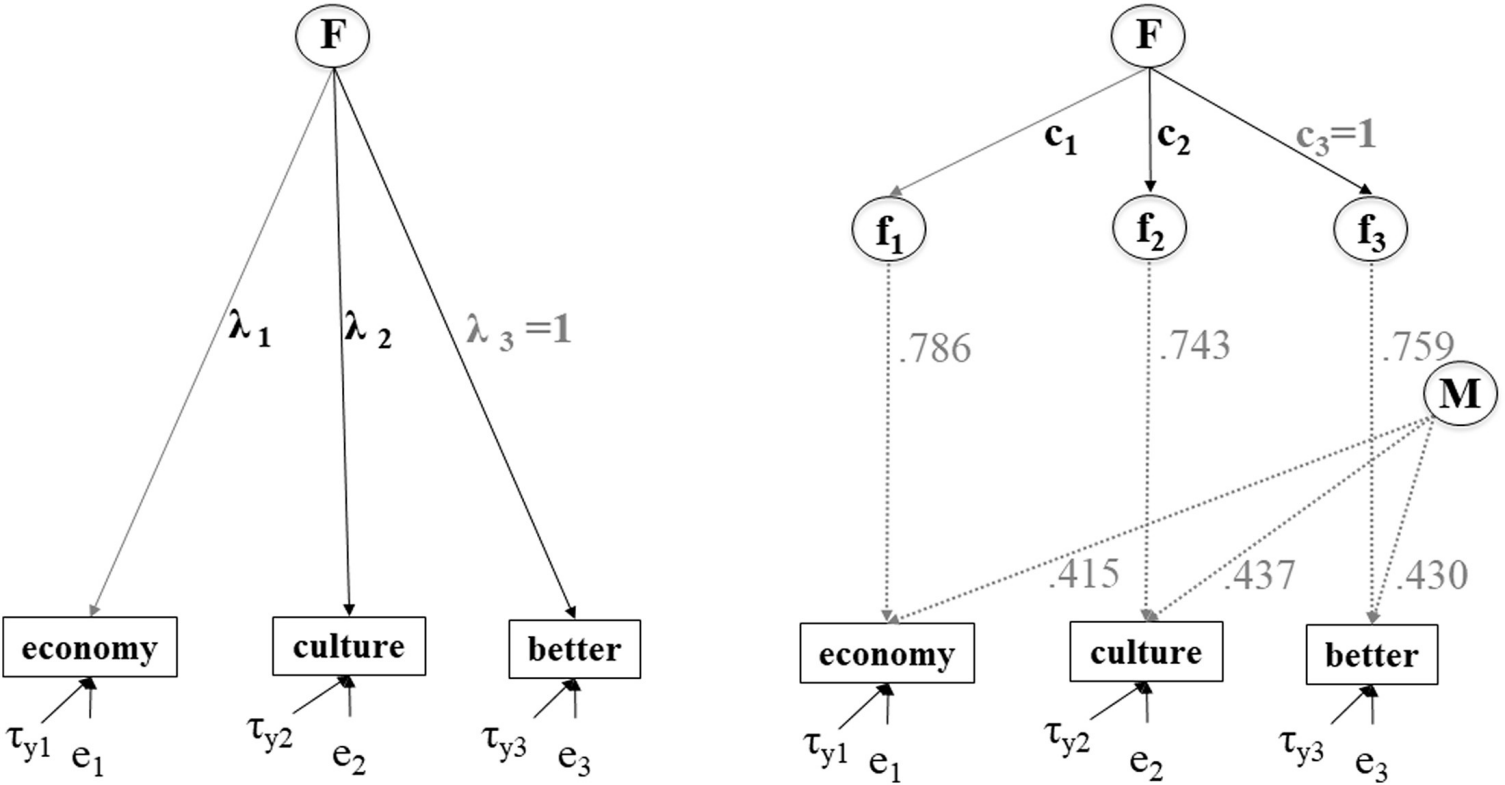
Models without and with correction for measurement error.

## 6. Analytical strategy

Our aim is to show the effect of correction for measurement error in invariance testing and we will therefore compare the findings of the different levels of invariance testing with and without correction for measurement error.

A factor model with three indicators is a just identified or saturated model which means that number of free parameters equals exactly the number of known values and hence there are zero degrees of freedom for testing. This means that we cannot test if the factor structure is the same across groups. Configural invariance must be assumed and the analysis starts with the test for metric invariance. However, the factor structure (configural invariance) is tested along with the equality restrictions of the loadings at the metric level. The measurement invariance approach we follow is bottom-up, meaning that the stricter test requires that the less strict level was achieved. At the metric invariance level, first we consider possible misspecified cross-loadings to evaluate the structural form of the model before focusing on the loadings’ equality restrictions across groups. Then, only if metric invariance was established, we test for scalar invariance by mainly looking for misspecified intercept equality constraints. We do not test for further levels of invariance as metric invariance allows for comparison of (unstandardized) relationships with other variables and scalar invariance for the comparison of latent means which is used in most substantive analyses.

Fig 3 illustrates the two different models for “Perceived ethnic threat” for the case of Austria. For identification, the loading of the indicator “better” (λ_3_ and c_3_) in all the groups is fixed to be 1, in what is also known as reference indicator parameterization. While other parameterizations are possible, this specification of the model has the advantage of producing an unstandardized solution which is particularly useful for measurement invariance testing [40]. In the model with correction for measurement error, further parameter constrains have to be introduced: the validity coefficient (v), reliability coefficients (r), and the method effect coefficients (m_i_) are fixed to the values obtained from SQP, the intercepts of the latent factors (F, f_i_, and M) as well as the unique components (u_fi_) are set to zero.

The models are estimated using the maximum likelihood estimator of Lavaan [41], an R package for structural equation modeling. We consider that our main latent variable (“Perceived Ethnic Threat”) is continuous, while it is measured with three observed indicators with 11 points answer scales each. Research has shown that items with 5 or more response categories can be treated as continuous with maximum likelihood estimators [see e.g. 42, 43]. For model evaluation and testing, we rely on the SemTools function *miPowerFit()* [44] which applies the local model testing procedure developed by Saris, Satorra, and van der Veld [45]. As known in the field of Structural equation modelling (SEM), the different global fit indices (GFI) are unequally sensitive for different misspecifications [see e.g. for a detailed overview 46]. Their cutoff values are derived from analyses of simulated data [47, see e.g. 48], being 0.06 or lower for RMSEA and 0.90 or higher for CFI, and they act as a statistics for hypothesis testing, i.e. if the critical value is exceeded, the entire model is rejected and if not, it is accepted. However, this way neither Type I (“false positive”) or Type II (“false negative”) errors, or their probabilities is controlled for. In face of this, Saris et al. [49] suggested taking the power of the test into account and argue against testing the model as a whole. A practical solution to this problem was later developed as a test for misspecifications of the model on the parameter level by using the modification index (MI) as test statistic for detection of misspecifications in combination with the expected parameter change (EPC) and the power of the test (46). This test is available for LISREL [50], for Mplus [51] and for R as SemTools function’ *miPowerFit()* [52]. The criterion for misspecifications must be set by the researcher. For this study, we aimed at detecting deviations of 0.1 in the loadings and approximately 5% of the scale length for the intercepts, i.e. a deviation of 0.6 in the intercepts given the 11-point scale. This test can lead to four different suggestions for decisions as presented in Table 3.

**Table 3:** Suggested decisions defined by the size of the modification index and power of the test.

	High power	Low power
**Significant MI**	Inspect Expected Parameter Change (EPC)	Misspecification present (m)
**Non significant MI**	No misspecification (nm)	Inconclusive (I)

The local fit testing decision procedure is extensively explained in van der Veld and Saris [53]. It should be highlighted that, as the authors explain, a certain number of misspecifications can occur by chance alone and it is not absolutely necessary to solve all indicated misspecifications. Our testing strategy is the following: at the metric level, we only consider misspecifications in the loadings or correlated errors. At the scalar level, we consider misspecified intercepts and loadings. We free the parameters where misspecifications are detected one by one, i.e. we first introduce one change to the model, estimate and test the model again, and then evaluate it once again using *MiPowerFit()*.

The same procedure is followed when evaluating the model corrected for measurement error. Because the measurement information was introduced with the predictions provided by SQP, we are not interested in the misspecifications that *miPowerFit()* indicates regarding the measurement part but only for the cognitive part of the model.

While we rely on local testing given the known problems of the global goodness-fit-indices (GFI), we still present GFI for readers’ convenience to evaluate the improvement of the models based on addressing the misspecifications. We present the root mean square error of approximation (RMSEA) and the change in comparative fit index (CFI) that was found to be one of the robust statistics for testing between-group invariance of CFA models [54]. However, there is no consensus about the global fit indices and its cutoff values. Chen [55] suggests a criteria for change of ≥.010 or .015 for RMSEA and of ≤-.005 or -.010 for CFI, while Rutkowski and Svetina [56] agree with this for scalar invariance tests but conclude from their analyses that change in CFI of -.02 and RMSEA of .03 were most appropriate for tests of metric invariance with large groups.

## 7. Results

### 7.1 Model with no correction for measurement error

Given that the model has one latent variable and three indicators, the model is just identified, i.e. there are no degrees of freedom to test the model for the configural level of invariance. The analysis starts therefore with the metric invariance model, meaning that the loadings are constrained to be equal across groups.

Evaluating the initial metric invariance model with the *miPowerFit()* function, it indicates a misspecification caused by a correlated error between the indicators *“better”* and “*culture”* in some groups. Although the problem of correlated errors in invariance testing is recognized and acknowledged, views on how to solve this problem divide the academic debate [57–59]. On the one side, scholars argue that correlated errors can and should be added to the model accordingly to the statistical criteria provided [57]. On the other side, allowing for correlated errors completely changes the model and requires therefore that theoretical reasons are behind the reasoning to allowing it or, as an alternative that the groups where this happens should be dropped from the analysis [59]. In what concerns the present study, as the aim is to test if the same model fits the data in the different country groups and in the absence of any strong theoretical reason for the presence of a correlated error, we consider that the measurement model for that group is different and consequently it cannot be compared to the remainder groups. With this reasoning in mind, the local fit testing shows misspecified correlated errors in the groups of France, Denmark and Estonia (see Appendix II). The most severe misspecified correlated error between the indicators *“better”* and “*culture”* is present in the country group of France and we proceed therefore without this group. We repeat the analysis thus with 18 country groups. The misspecifications are still present in the groups of Denmark and Estonia. Focusing on the EPC and Modification Index, the most severe misspecification is shown to be in Denmark country group. Just as before, we take this group out of the sample and repeat the analysis. Following this same procedure, the country group of Estonia was also excluded from the sample, which then consists of 16 country groups.

After these groups are taken out of the analysis because of the correlated errors, no further relevant misspecifications are found by *miPowerFit()* and the global fit indices also suggest an improvement, although CFI is already suggesting that the data fits the initial model, being >0.9, RMSEA only indicates this for the final metric model, being < .06 (Table 4) Thus, our analysis shows that the model is metric invariant for 16 out of the 19 countries included in the analysis. The unstandardized loadings for the indicators are presented in Table 5. We report the unstandardized solution because following Brown [40] the analysis itself is based on unstandardized variables and completely standardized values are potentially misleading.

**Table 4:** Global fit indices for model without correction for measurement error.

	Initial metric model	Final metric model	Scalar model
**Country groups**	**19**	**16**	**16**
**Chi-square**	333.456	179.506	3010.492
**Degree of freedom**	36	30	60
**P-value**	0.000	0.000	0.000
**RMSEA**	0.069	0.053	0.165
**CFI**	0.993	0.996	0.912

RMSEA = Root Mean Square Error of Approximation, CFI = Comparative Fit Index.

**Table 5:** Unstandardized loadings after metric invariance test for the model without correction for measurement error.

Loadings	Estimate	Std. error	95% CI
“economy”	.940	.007	[0.926; 0.955]
“culture”	1.035	.008	[1.020; 1.051]
“better”	1.000	-	-

Three excluded countries: Estonia, Denmark, France.

In face of the metric invariance results, we can now proceed to test for scalar invariance which implies that in addition to the loadings also the intercepts are constrained to be equal across groups. As before the model is evaluated using *miPowerFit()* which indicates no misspecification of intercepts and loadings. The intercepts of the two indicators which were estimated are presented in Table 6. We can conclude from the analysis that the groups France, Denmark and Estonia are not metric invariant and therefore cannot be compared with the remainder groups. The global fit indices, RMSEA < .06 and CFI < .9, also indicate that the model fits the data (Table 4). In Table 6 we present the unstandardized intercepts after scalar invariance testing.

**Table 6:** Unstandardized intercepts after scalar invariance test for the model without correction for measurement error.

Intercepts	Estimate	Std. error
“economy”	0.480	0.038
“culture”	0.606	0.040

Three excluded countries: Estonia, Denmark, France.

### 7.2 Model with correction for measurement error

Just as before, we start with the metric invariance model, constraining the loadings to be equal across groups. In addition, here we fix the measurement part of the model to the estimates obtained from SQP. We evaluate the results with *miPowerFit()* and check for misspecifications regarding the model parameters, focusing first on the structure and then on the constrained loadings. We encounter a large number of misspecified parameters and set them to be free one by one, always estimating the model again after each of these operations and evaluating the results again with *miPowerFit()*. Following this procedure, we had to free the “*culture*” indicator loadings constrained to be equal in the groups of France and Finland (see Appendix II). As for the “*economy*” indicator, the groups of Denmark and Estonia also have misspecified loadings. The estimates are presented in Table 7.

**Table 7:** Unstandardized loadings after metric invariance test for the model corrected for measurement error.

Loadings		Estimate	Std. error	95% CI
“economy”	17 comparable country groups	0.932	0.007	[0.918; 0.946]
	Denmark	0.739	0.025	[0.690; 0.787]
	Estonia	0.726	0.033	[0.660; 0.790]
”culture”	17 comparable country groups	1.036	0.008	[1.020; 1.051]
	France	1.225	0.025	[1.176; 1.273]
	Finland	0.872	0.027	[0.820; 0.924]
“better”	-	1.000	-	-

The results indicate that all the groups are at least partially metric invariant even though not all the groups can be directly compared. Furthermore, what is also relevant is that the metric invariance testing results with correction for measurement error gives no indication of misspecified correlated errors. Consequently, as all the groups are at least partially metric invariant, no group had to be excluded. This allows considering that the change in the GFI also fit the previously discussed criteria. This means that they can be used to study the relationships of the “Perceived ethnic threat” concept with other variables across the country groups in a latent variable model.

Moving forward with the analysis, the same procedure was followed in the subsequent step of the invariance analysis, testing for scalar invariance. The model constraining the loadings and the intercepts to be equal between groups was estimated and evaluated using *miPowerFit()*. The metric invariant parameters are freed from the equality constraint. The changes in global-fit-indices of the metric and scalar invariance models can be found in Table 8.

**Table 8:** Global fit indices for models with correcting for measurement error.

Model	Chi-square	Δ Chi-square	df	Δ df	P-value for chi-square difference test	RMSEA	Δ RMSEA	CFI	Δ CFI
Initial metric model^1^	330.661		36			0.068		0.993	
Final metric model^2^	149.592	181.069	32	4	0.000	0.046	0.022	0.997	-0.004
Initial scalar model^3^	2676.873	-2527.281	64	-32	0.000	0.152	-0.106	0.935	0.062
Final scalar model^4^	1759.987	916.886	62	2	0.000	0.125	0.027	0.958	-0.023

^1^ all loading set equal across groups,

^2^ partial metric invariance: loadings of “economy” for Denmark and Estonia as well as of “culture” for Finland and France were not invariant,

^3^ all intercepts with the exception of Denmark, Estonia, Finland, and France were constrained to be equal,

^4^ partial scalar invariance: intercepts of “economy” for Denmark, Estonia, Austria, and Sweden as well as of “culture” for Finland and France were not invariant.

RMSEA = Root Mean Square Error of Approximation,

CFI = Comparative Fit Index,

Δ = change of chi-square difference test for nested model and change of RMSEA and CFI.

Just as before, the misspecified parameters are freed from the model restrictions one by one until no more relevant misspecifications are indicated by *miPowerFit()*. In addition to the deviant loadings of France, Finland, Estonia and Denmark detected in the metric analysis, now we have to free one parameter for the groups of Austria and Sweden. The final results, also supported by the GFI change criteria, show that all the groups are at least partially scalar invariant even though, as before, not all the groups can be compared with each other. The loading of the indicator “*economy*” was deviant in the groups of Denmark, Estonia, Austria and Sweden. These groups can be compared with all the other groups with the exception of France and Finland which had a deviant loading in the “*culture*” indicator. Table 9 presents the intercepts of the indicators in all country groups.

**Table 9:** Unstandardized intercepts after scalar invariance test for the model corrected for measurement error.

Intercepts		Estimate	Std. error
“economy”	15 comparable country groups	0.300	0.037
	Denmark	0.918	0.158
	Estonia	1.520	0.155
	Austria	1.185	0.050
	Sweden	-0.651	0.059
“culture”	17 comparable country groups	0.602	0.037
	Finland	2.301	0.152
	France	-0.355	0.119

We can conclude from the measurement invariance analysis with correction for measurement error that metric and scalar invariance holds for all country groups. However, several groups are partially invariant and cannot be directly compared. Namely, this is the case of Denmark, Estonia, Austria and Sweden that should not be compared with the groups of Finland and France.

Even though there are still an important number of groups which are only partially measurement invariant, it is clear that applying correction for measurement error improved the results for comparability across countries. The fit indices were consistently better after correction for measurement error and the misspecifications due to correlated errors also disappear. While in the model without correction the groups of France, Denmark and Estonia are not metric invariant, when we correct for measurement error these same groups are then partially invariant. In other words, those three country groups that were considered not comparable can still be meaningfully compared after correction for measurement error and taking the limitations of partial invariance into account.

Now that we know which groups are full scalar invariant with and without correction for measurement error, we can also check whether there are significant differences in the latent means scores after correction. Scholars are often interested in comparing country means, either as composite scores or latent means. In Table 10 we present, for illustration, the latent mean score and its rank by group estimated with and without correction for measurement error. We can see that the ranking is different. For example, without correction for measurement error, the Slovenian group was the third lowest mean raking group, while after correction it ends up being the fifth. The Netherland group, on the other hand, ranked 10 before and 8 after correction for measurement error. This illustration serves to show that measurement error is a factor that left unaccounted can bias latent mean score comparisons.

**Table 10:** Country latent means and rankings of “Perceived ethic threat”.

Country	Latent Mean Score^1^	Uncorrected Mean Ranking	Latent Mean Score^1^	Corrected Mean Ranking
Belgium	4.730	7	6.250	6
Germany	4.631	5	6.129	4
Ireland	5.609	12	7.652	12
Netherlands	5.166	10	6.491	8
Norway	5.163	9	6.889	10
Poland	5.801	13	7.887	13
Portugal	4.492	4	5.963	3
Slovenia	4.393	3	6.146	5
Slovakia	4.664	6	6.444	7
Spain	5.147	8	6.937	11
Switzerland	5.414	11	6.805	9
Ukraine	4.103	1	5.688	1
United Kingdom	4.233	2	5.700	2

^1^ The scale of the latent variables ranges from 0 to 11 as the loading of “better” was fixed to 1.

## 8. Conclusions

In this study we addressed two issues: The first was to examine if the concept of “perceived ethic threat” of immigration from the ESS was measurement invariant across the different country groups. The second was to show to what extent correction for measurement error impacts measurement invariance results.

Regarding the first question, we have shown that the concept of “perceived ethic threat” without correction for measurement error reaches only at best partial invariance within the countries that took part in the ESS round 3. Following the results of the analysis without correction for measurement error, Denmark, Estonia and France cannot be compared with the rest of the groups. Disentangling the cognitive from the measurement process by correcting for measurement error, we find that Denmark, Estonia and France do not need to be excluded, as partial scalar invariance was established. The same holds for Austria, Sweden and Finland. This implies that the measurement invariance conclusions of the uncorrected model were driven by the measurement method used in these groups, i.e. the measurement part, rather than the cognitive part related to the “perceived ethic threat” concept. Even though more groups can be meaningfully compared after correction for measurement error, a note of caution regarding the use of simple composite scores is necessary. As only partial invariance was achieved, researchers interested in the latent mean differences are advised to use latent variable models as the creation of simple composite scores can lead to biased conclusions [60].

As for the second question, we hope to have been successful in showing clearly that measurement error can affect measurement invariance analysis results. The invariance testing procedure without correction for measurement error indicated that three countries, namely Denmark, Estonia and France had to be excluded due to the presence of correlated errors. In contrast, after correction for the measurement part, these groups were proven to be partially invariant instead of non-invariant. This means that not correcting for measurement error adds to the risk of excluding a group because of measurement error and not because of the substantial issue of interest. In the case presented here, we would not be able to compare unstandardized relationships and the latent means of Denmark, France and Estonia regarding “perceived ethic threat” with other European countries.

This study has also limitations. Amongst these, the most significant is that to correct for measurement error we rely on the SQP software which is only available at the moment for European countries and languages. Researchers working with data from other regions or languages of the world cannot depend on this resource, shortening the possibilities to address the issue of measurement error on measurement invariance. Moreover, SQP is an ongoing research project continuously updated with more experiments in its underlying meta-analysis. This obviously affects the quality predictions which as more information is used, increase their precision. In the current version the prediction has a R^2^ of 0.84 and 0.65 for the validity (v) and reliability coefficient (r) logits, respectively [35]. This means that SQP does not yet predict the measurement quality perfectly. However, even though SQP has in itself limitations, in the absence of any other available source of measurement quality, it allows correcting for measurement error in survey data.

On the other hand, this study also shows that while there is research using European data regarding the invariance property of the concept “perceived ethnic threat”, there are reasons to believe that correction for measurement error can change some of the conclusions. This is particularly significant because we use ESS data, most likely one of the cross-national surveys that dedicates the most resources in assuring cross-cultural comparability, and nonetheless measurement error still occurs. To conclude, correction for measurement error should be increasingly regarded as valid avenue to improve both survey and substantial research standards.
